# Balanced crystalloids for intravenous fluid therapy in critically ill and non-critically ill patients: A protocol for systematic review and network meta-analyses

**DOI:** 10.1097/MD.0000000000013683

**Published:** 2018-12-21

**Authors:** Peifen Ma, Bo Wang, Jun Zhang, Xiping Shen, Liping Yu, Xinman Dou

**Affiliations:** a Department of Nursing, Lanzhou University Second Hospital; b School of Nursing, Lanzhou University; c Department of Nursing, Rehabilitation Center Hospital of Gansu Province; d School of Nursing, Gansu University of Chinese Medicine; e Institute of Epidemiology and Health Statistics, School of Public Health, Lanzhou University, Lanzhou, China.

**Keywords:** balanced crystalloids, critically ill patients, intravenous fluid therapy, network meta-analysis, non-critically ill patients

## Abstract

**Background:**

The balanced crystalloids have become a substitute for saline for fluid resuscitation. Some studies have investigated the clinical effect and adverse event of differently balanced crystalloids, but they have no consistent conclusions. This study aims to assess and compare the effect of differently balanced crystalloids for intravenous fluid therapy in critically ill and non-critically ill patients using network meta-analysis (NMA).

**Methods:**

Electronic databases including PubMed, EMBASE, Cochrane Library, Web of Science, Clinical Trials.gov, and the International Clinical Trials Registry Platform (ICTRP) will be searched from inception to April 2018. We will include randomized controlled trials (RCTs) that reported the effect and adverse event of balanced crystalloids. Risk of bias assessment of the included RCTs will be conducted according to the Cochrane Handbook 5.1.0. A Bayesian NMA will be performed using R software. GRADE will be used to explore the quality of evidence.

**Results:**

The results of this NMA will be published in a peer-reviewed journal.

**Conclusion:**

This NMA will summarize the direct and indirect evidence to assess the effect of differently balanced crystalloids.

**Ethics and dissemination:**

Ethics approval and patient consent are not required as this study is an NMA based on published studies.

**PROSPERO registration number:**

CRD42018093818.

## 1 Introduction

Intravenous isotonic crystalloid is one of the most commonly used medications in daily medical practice, especially in hospital wards, intensive care units (ICUs), emergency departments, and operating rooms.^[1–3]^ In all crystalloids, isotonic saline is most commonly used for fluid resuscitation.^[3–5]^ In the United States, more than 200 million liters of saline are used each year.^[6]^ However, previous studies have shown that intravenous saline may lead to hyperchloremic metabolic acidosis,^[7]^ acute kidney injury,^[8]^ death,^[9,10]^ and impair a patient's ability to recover from severe illness.^[11]^ The balanced crystalloids such as lactated Ringer's solution and Plasma-Lyte solution have electrolyte compositions closer to plasma.^[12,13]^ Therefore, in recent years, more and more balanced crystalloids have become a substitute for saline for the recovery of patients with surgery, trauma and diabetic ketoacidosis.^[9,14–16]^ Some observational studies have shown that the use of balanced crystalloids can reduce acute kidney replacement therapy, kidney injury, and mortality.^[8,9,17]^ But some studies suggested that there was no statistical difference in these outcomes between patients receiving balanced crystalloids and receiving saline.^[11,18]^

Recently, many meta-analyses compared the effect, renal injury incidence, and mortality of different balanced crystalloids and saline for critically ill and non-critically ill patients, but there are considerable differences in conclusions.^[12,19–21]^ In addition, these studies are traditional meta-analyses, and it is difficult to assess the effects of more than 2 interventions through traditional meta-analysis. Network meta-analysis (NMA) has the advantage of allowing indirect comparisons of multiple interventions for estimation and ranking their orderings even though direct head-to-head comparison studies are lacking.^[22]^ Thus, it is important to integrate these studies using NMA. The objectives of this NMA are to investigate the clinical effect of differently balanced crystalloids for intravenous fluid therapy in critically ill and non-critically ill patients and to find the best-balanced crystalloid by comparing the effects of differently balanced crystalloids.

## 2 Methods

### 2.1 Study registration

The protocol of this study has been registered on International Prospective Register of Systematic Reviews (PROSPERO). The registration number is CRD42018093818.

### 2.2 Eligibility criteria

#### 2.2.1 Type of studies

Randomized controlled trials (RCTs) compared any type of balanced crystalloid with non-balanced crystalloids used for intravenous fluid therapy in critically ill and non-critically ill patients will be included. There are no language restrictions.

#### 2.2.2 Patients

We will include patients with acute disease or undergoing major surgery and at least 1 intervention group received balanced crystalloids.

#### 2.2.3 Interventions

All types of balanced crystalloids used for intravenous fluid therapy will be included, including Ringer's lactate, Ringer's solution, Plasmalyte A, compound electrolyte solution, and so on. There are no limitations on the method of administration, the number, dosage, and duration of treatments.

#### 2.2.4 Outcomes

The primary outcomes are major adverse kidney events, death, and new renal replacement therapy. The secondary outcomes are hemodynamic instability, renal dysfunction, risk of sepsis, myocardial infarction, stroke, duration of ICU, and hospital stay. RCTs report at least 1 primary outcome will be included.

#### 2.2.5 Other criteria

Studies will be excluded if any of the following characteristics are present:

(1) studies enrolling only healthy volunteers or blood donors;
(2) administration of fluid solely for the purposes of a planned anesthetic procedure including spinal or epidural anesthesia, acute normovolemic hemodilution, hypervolemic hemodilution or priming of a cardiopulmonary bypass circuit without subsequent intraoperative or postoperative use;
(3) administration of fluid solely for volume therapy (hemodilution) following ischemic stroke or subarachnoid hemorrhage. There are no limitations on age, gender, race, or nationality.

### 2.3 Data sources

We will conduct a computerized search of the electronic databases PubMed, EMBASE, Cochrane library, and Web of Science for RCTs from inception to April 2018. We will also search for Clinical Trials.gov and the International Clinical Trials Registry Platform (ICTRP) for ongoing trials. Furthermore, we will retrieve the references of included studies and relevant systematic reviews and will contact experts for any additional relevant evidence.

### 2.4 Search strategy

The key search terms are balanced crystalloids, Ringer's lactate, Ringer's solution, Plasmalyte A, compound electrolyte solution, normal saline, physiologic salt solution, hypertonic saline solution, dextran, hydroxyethylated starches, starch, albumin, and succinylated gelatin. Search strategy of PubMed was as follows:

- #1 “Dextrans”[Mesh]
- #2 Dextran∗[Title/Abstract] OR Hemodex[Title/Abstract] or Hyskon[Title/Abstract] or Infukoll[Title/Abstract] or Macrodex[Title/Abstract] or Polyglucin[Title/Abstract] or Promit[Title/Abstract] or Rheodextran[Title/Abstract] or Rheoisode[Title/Abstract] or Rheomacrodex[Title/Abstract] or Rheopolyglucin[Title/Abstract] or Rondex[Title/Abstract] or Saviosol[Title/Abstract]
- #3 “Hydroxyethyl Starch Derivatives”[Mesh]
- #4 Hydroxyethylated Starches[Title/Abstract] or Hemohes[Title/Abstract] or Elohes[Title/Abstract] or Hespan[Title/Abstract] or Hetastarch[Title/Abstract] or Pentafraction[Title/Abstract] or Pentaspan[Title/Abstract] or Pentastarch[Title/Abstract] or Plasmasteril[Title/Abstract] or HAES-steril[Title/Abstract] or Hydroxyethyl Starch[Title/Abstract] or Starch∗[Title/Abstract]
- #5 “Albumins”[Mesh]
- #6 C-Reactive Protein[Title/Abstract] or Avidin[Title/Abstract] or Ricin[Title/Abstract] or Technetium Tc 99m Aggregated[Title/Abstract] or Albumin∗[Title/Abstract]
- #7 “succinylated gelatin” [Supplementary Concept]
- #8 succinylated gelatin[Title/Abstract] or gelofusin[Title/Abstract]
- #9 “Ringer's lactate” [Supplementary Concept]
- #10 Lactated Ringer's Solution[Title/Abstract] or Ringer's lactate[Title/Abstract] or Hartmann's solution[Title/Abstract]
- #11 “Ringer's solution” [Supplementary Concept]
- #12 Ringer's solution[Title/Abstract] or Isotonic Solutions[Title/Abstract]
- #13 “Saline Solution, Hypertonic”[Mesh]
- #14 Hypertonic Solution[Title/Abstract] or Hypertonic Saline Solution[Title/Abstract]
- #15 “Plasmalyte A” [Supplementary Concept] or “Ringerfundin” [Supplementary Concept] or “Hanks Balanced Salt Solution” [Supplementary Concept] or “PentaLyte” [Supplementary Concept]
- #16 Plasma-Lyte A[Title/Abstract] or balanced crystalloids[Title/Abstract] or compound electrolyte solution[Title/Abstract] or multiple electrolytes injection[Title/Abstract]
- #17 or /1–16
- #18 “Death”[Mesh] or “Mortality”[Mesh] or “Survival Rate”[Mesh]
- #19 Death[Title/Abstract] or Mortality[Title/Abstract] or Survival Rate[Title/Abstract]
- #20 #18 or #19
- #21 “Clinical Trials, Phase II as Topic”[Mesh] or “Clinical Trials, Phase III as Topic”[Mesh] or “Clinical Trials, Phase IV as Topic”[Mesh] or “Controlled Clinical Trials as Topic”[Mesh] or “Randomized Controlled Trials as Topic”[Mesh] or “Intention to Treat Analysis”[Mesh] or “Pragmatic Clinical Trials as Topic”[Mesh] or “Clinical Trials, Phase II”[Publication Type] or “Clinical Trials, Phase III”[Publication Type] or “Clinical Trials, Phase IV”[Publication Type] or “Controlled Clinical Trials”[Publication Type] or “Randomized Controlled Trials”[Publication Type] or “Pragmatic Clinical Trials as Topic”[Publication Type] or “Single-Blind Method”[Mesh] or “Double-Blind Method”[Mesh]
- #22 random∗[Title/Abstract] or blind∗[Title/Abstract] or singleblind∗[Title/Abstract] or doubleblind∗[Title/Abstract] or trebleblind∗[Title/Abstract] or tripleblind∗[Title/Abstract]
- #23 #21 or #22
- #24 #17 AND #20 and #23

### 2.5 Study selection

We will obtain the titles and abstracts of relevant literature from the database search techniques outlined in the search strategies. Records will be managed by EndNote X7 software. Two reviewers will independently screen and categorize all related articles, and the full-texts of any potentially eligible studies will be retrieved independently by the same authors and examined to determine whether they meet the inclusion criteria. Multiple submissions or duplicate publications will be compared, and the more detailed 1 will be retained. The reasons for the exclusion of any articles will be recorded, and, if necessary, an additional author will be consulted to resolve any controversial issues.

Two reviewers will independently extract the required data from the studies selected for inclusion, using the double-entry method to verify the accuracy of the data. Any discrepancies arising between them over the extracted data will be resolved by consensus, and the data will be rechecked by a third investigator.

### 2.6 Risk of bias assessment

Two authors will independently assess the methodological quality of the included studies, using the Cochrane risk of bias assessment tool which includes reference to the following items: sequence generation, allocation concealment, blinding of participants, personnel and outcomes assessors, incomplete outcome data, selective outcome reporting, and other sources of bias.

Each item will be assessed as being of “low risk”, “high risk”, or “unclear” risk of bias. Any disagreement will be discussed with another reviewer, and the quality of evidence assessment will be used to assess the quality level of the evidence.

### 2.7 Statistical analysis

We will provide a narrative synthesis of the findings of the included studies based on the type of intervention, the target population characteristics, the type of outcome, and the intervention content. We will also provide summaries of the intervention effects for each study by calculating risk ratios (for dichotomous outcomes) or standardized mean differences (for continuous outcomes). The results will be pooled using a random-effects meta-analysis, and we will calculate 95% confidence intervals and bilateral *P* values for each outcome. We will consider an *I*^*2*^ value greater than 50% as being indicative of substantial heterogeneity.

For the NMA, direct and indirect evidence from all relevant studies will be integrated. The NMA will be conducted in a Bayesian framework using a random effects model and the GeMTC package, which recalls JAGS in the R project. In this method, non-informative prior distributions and Markov chain Monte Carlo (MCMC) simulations will be used, and 4 parallel chains will be applied, with 50000 samples being obtained after a 20000-sample burn-in in each chain. Additionally, the convergence will be assessed using the Brooks-Gelman-Rubin diagnostic, and inconsistency, statistical disagreement of direct and indirect evidence will be assessed using the node split method. We will regard *P* <.05 as being indicative of significant inconsistency, and we will also rank each treatment according to the probability that 1 is superior to the other.

If the necessary data are available, subgroup analyses will be done for different types of participants by age, types of colloid, clinical settings.

### 2.8 Quality of evidence

The quality of evidence for the main outcomes will be assessed using the Grading of Recommendations Assessment, Development, and Evaluation,^[23]^ which contains 4 levels: high level, moderate level, low level, and very low level. Flaws in study design, inconsistency, imprecision, indirectness, and publication bias will be investigated.

## 3 Discussion

The efficacy and of balanced crystalloids have been assessed mainly using traditional meta-analysis. To the best of our knowledge, there are no network meta-analyses comparing the clinical effect of differently balanced crystalloids for intravenous fluid therapy in critically ill and non-critically ill patients. This leads to a lack of reliable evidence to guide the choice of balanced crystalloids in clinical practice. This NMA will summarize the direct and indirect evidence to assess the effect of differently balanced crystalloids. Furthermore, we will assess the quality of evidence using the GRADE framework. We hope that the results of this NMA will help clinicians and caregivers make more appropriate choices when using balanced crystalloids.

## Author contributions

PM, BW, and XD planned and designed the study. JZ and LY tested the feasibility of the study. XS and XD provided methodological advice, polished and revised the manuscript. PM, BW, and XD wrote the manuscript. All authors approved the final version of the manuscript.

**Conceptualization:** Peifen Ma, Bo Wang.

**Investigation:** Peifen Ma.

**Methodology:** Xiping Shen, Xinman Dou.

**Project administration:** Xinman Dou.

**Resources:** Bo Wang, Jun Zhang, Liping Yu.

**Software:** Bo Wang, Xiping Shen.

**Supervision:** Xinman Dou.

**Validation:** Jun Zhang, Liping Yu.

**Visualization:** Peifen Ma, Xinman Dou.

**Writing – original draft:** Peifen Ma, Bo Wang.

**Writing – review & editing:** Xinman Dou.
